# Depressive symptoms in patients diagnosed with benign prostatic hyperplasia

**DOI:** 10.1007/s11255-015-0920-5

**Published:** 2015-02-12

**Authors:** Barbara Pietrzyk, Magdalena Olszanecka-Glinianowicz, Aleksander Owczarek, Tomasz Gabryelewicz, Agnieszka Almgren-Rachtan, Andrzej Prajsner, Jerzy Chudek

**Affiliations:** 1 Pathophysiology Unit, Department of Pathophysiology, Medical Faculty in Katowice, Medical University of Silesia, 18 Medyków Street, 40-752 Katowice, Poland; 2 Health Promotion and Obesity Management Unit, Department of Pathophysiology, Medical Faculty in Katowice, Medical University of Silesia, 18 Medyków Street, 40-752 Katowice, Poland; 3 Division of Statistics, Department of Instrumental Analysis, Faculty of Pharmacy and Laboratory Medicine in Sosnowiec, Medical University of Silesia, Ostrogórska Street 30, 41-200 Sosnowiec, Poland; 4 Department of Neurodegenerative Disorders, Mossakowski Medical Research Centre Polish Academy of Sciences, Pawińskiego Street 5, 02-106 Warsaw, Poland; 5 Department of Pharmacovigilance, Europharma Rachtan Co.Ltd, Krzywa Street 6, 40-061 Katowice, Poland; 6 Department of Urology, Medical Faculty in Katowice, Medical University of Silesia, Medyków Square 1, 41-221 Sosnowiec, Poland

**Keywords:** Depressive symptoms, Benign prostatic hyperplasia, Erectile dysfunction, Risk factors, Cohort study

## Abstract

**Background:**

Symptoms of depression are common in patients diagnosed with benign prostatic hyperplasia (BPH) and are usually a reaction to deterioration of health, severity of lower urinary tract symptoms, and erectile dysfunction. The aim of this observational study was to evaluate the prevalence of depressive symptoms in patients diagnosed with BPH and factors affecting their occurrence in a large Polish cohort.

**Patients and methods:**

Four thousand thirty-five men (4,035) diagnosed with BPH participated in the survey (age 65 ± 8 years). The occurrence of symptoms of depression was assessed using the Beck depression inventory, severity of lower urinary tract symptoms (LUTS) on the basis of the international prostate symptoms score, and erectile dysfunction using the international index of erectile function (IIEF-5).

**Results:**

Depressive symptoms were found in 22.4 % of patients (mild in 20.8 % and moderate/severe in 1.6 %). Erectile dysfunction was found in 71.9 % of patients. Monotherapy for BPH was prescribed to 50.9 % of patients (mostly ARA—selective α1-selective alpha-adrenolytic—47.5 %), while polytherapy (ARA with a 5-alpha reductase inhibitor—5αRI) to 47.9 %. Logistic regression analysis showed a bidirectional relation between the occurrence of depressive symptoms and erectile dysfunction. The occurrence of both depressive symptoms and erectile dysfunction was related to severity of LUTS, nocturia, the use of 5αRI, comorbidity, and sedentary life style.

**Conclusions:**

Prevalence of depressive symptoms in patients diagnosed with BPH is associated with severity of LUTS, erectile dysfunction, nocturia, BPH pharmacotherapy (5αRIs), sedentary life style, and comorbidities including obesity.

## Introduction

The prevalence of benign prostatic hyperplasia (BPH) increases gradually from the age of 50–80 years old at an incidence of 80 % [1]. Lower urinary tract symptoms (LUTS) affect about 70 % of men aged 80 years, and more than half of them seek medical consultation due to symptomatic BPH [2]. Considering the above data, it can be assumed that in Poland, approximately two million men suffer from LUTS related to BPH. However, there are no reliable data concerning the prevalence of BPH and LUTS related to BPH in the general Polish population. The results of the PolSenior study have shown that one in every four men aged 65 years and over is treated for BPH [3].

During the last decade, an association between the occurrence of LUTS (frequent urination, urgent urination, and weakened urine flow) related to BPH and sexual dysfunction, including libido, erectile, and ejaculation disturbances, as well as decreased satisfaction with sexual life has been described [4–6]. In men with severe LUTS, the risk of ejaculation and erectile dysfunction is doubled and the risk of painful ejaculation is sixfold greater than in men without LUTS [8–10]. Some 83 % of men 50 years or older maintain sexual activity. Erectile dysfunction (ED) decreases self-esteem and has a negative impact on relationships [4, 11] which may be attributed to depressive symptom (DSs) development. It was also shown that ED may increase the risk of attempted suicide in men [11].

Additional risk factors for DSs development in subjects diagnosed with BPH are sleep disturbances related to nocturia, impairment of daily functioning caused by LUTS severity, as well as fear of developing prostate cancer and surgical procedures [5, 7].

It has also been suggested that adverse effects of drugs used for BPH treatment and surgical procedures are risk factors for both ED and DSs development. Use of inhibitors of 5-α reductase (5αRI) may be a cause of ED [12, 13], and selective α_1_-adrenergic receptor antagonists (ARA) may impair ejaculation [14]. Contradictorily, it was also found that the quality of life significantly improve in patients treated for BPH with 5αRI and ARA during the 4-year follow-up [15]. The decrease of LUTS severity and discomfort related to LUTS as well as improvement of the quality of life and increased satisfaction with life was also shown after 6 months of BPH treatment with 5αRI [16].

The prevalence of DSs and their influence in subjects diagnosed with BPH in Poland has not been assessed, yet. Therefore, the aim of this observational study was to evaluate the prevalence of depressive symptoms in patients diagnosed with BPH and factors influencing their occurrence in a large Polish cohort.

## Methods

### Patients and study design

In this observational survey, 4,035 men diagnosed with BPH were interviewed nationwide from November 2012 to Jun 2013 by 206 urologists from specialist outpatient clinics. Polish doctors participating in the study were recruited by medical representatives, and each of them conducted questionnaire interviews with a group of 15–30 consecutive patients diagnosed with BPH referred to the clinic.

The study procedures were in accordance with the ethical standards and the Helsinki Declaration of 1975, as revised in Seoul during 2008. As the questionnaire-based survey did not fulfil the criterion of a medical experiment, Bioethics Committee approval was not required.

The inclusion criterion was age over 40 years and BPH diagnosis. The exclusion criteria included dementia, deafness, active psychiatric disorders, mood disorders in medical history, and diagnosed prostate cancer. PSA was a part of routine clinical management, but was not reported. BPH diagnosis was based on medical history, the presence of LUTS, and results of DRE (digital rectal examination) tests and sonography. Characteristics of the surveyed population are summarised in Table 1.

**Table 1:** Baseline characteristics of the study group—4,035 patients diagnosed with benign prostatic hyperplasia (BPH)

Age [years]	65 ± 8 (range 40–92)
Age groups [n(%)]	
≤60 years	1,113 (27.6)
61–80 years	2,818 (69.8)
>80 years	104 (2.6)
Place of residence [n(%)]	
Rural areas	447 (11.1)
City with population <50,000	920 (22.8)
City with population 50,000–200,000	1,678 (41.6)
City with population >200,000	990 (24.5)
Education [n(%)]	
Primary	331 (8.2)
Vocational	1,393 (34.7)
Secondary	1,600 (39.6)
Higher	711 (17.5)
Marital status [n(%)]	
Married	2,975 (73.8)
Widowed/single	1,060 (26.2)
Labour activity [n(%)]	
Professionally active	1,758 (43.7)
Unemployed	172 (4.3)
Annuity	376 (9.4)
Pension	1,711 (42.5)
Other	8 (0.1)
Nutritional status [n(%)]	28.4 ± 3.8
Normal weight	572 (14.2)
Overweight	2,297 (56.9)
Obese	1,166 (28.9)
Waist circumference [cm]	96 ± 12
Visceral obesity [n(%)]	2,147 (53.5)
Physical activity [n(%)]	
Less than 30 min a day	2,671 (66.2)
30–60 min a day	995 (24.6)
More than 60 min a day	369 (9.2)
Alcohol consumption [n(%)]	
Frequent (at least three times a week)	760 (18.8)
Seldom	2,360 (58.5)
No	915 (22.7)
Cigarettes smoking	
Currently [n(%)]	1,203 (29.8)
The extent of exposure [pack-years]	24.0 ± 12.8
In the past [n(%)]	991 (24.5)

The questionnaire using in this study included demographic data (age, education level, place of residence, marital status, source of income), anthropometric measurements (body mass, height and waist circumference), lifestyle data (smoking, alcohol consumption, physical activity), and medical history (duration of BPH, duration of LUTS, pharmacotherapy used in treatment of BPH, pharmacotherapy used in treatment of ED, severity of LUTS before using the current pharmacotherapy, current severity of LUTS, past transurethral resection of the prostate—TURP, and comorbidities).

The occurrence of DSs was assessed using the Beck Depression Inventory (BDI) in Polish version [17], severity of LUTS on the basis of the international prostate symptoms score (IPSS) and ED using the international index of erectile function (IIEF-5). The Polish versions of IPSS and IIEF-5 questionnaires were validated by the Polish Society of Urology.

### Data analysis

The requisition of data was entered automatically with a specific form (Microsoft Office Access). Patients records (*N* = 53) with the diagnosis of prostate cancer were initially excluded. The percentage of missing data was less than 3 %, and those entries were not removed from the analysis, as missing data were at random.

Nutritional status was assessed on the basis of BMI according to WHO criteria (underweight was diagnosed with values <18.5 kg/m^2^, overweight 25–29.9 kg/m^2^ and obesity ≥30 kg/m^2^) [18]. Visceral obesity was diagnosed by measuring waist circumference according to the IDF criteria for Caucasians (≥94 cm for men) [19].

The occurrence of depressive symptoms was scored on the results of BDI: 12–26 points—mild and >26 points—moderate and severe group [20]. The severity of LUTS was classified on the basis of the IPSS scale as: mild (0–7 pts.), moderate (8–19 pts.), and severe (20–35 pts.) [21]. ED was diagnosed at the values of the IIEF-5 ≤21 pts. [22].

### Statistical analysis

Statistical analysis was performed using the STATISTICA 10.0 PL software package (StatSoft Krakow, Poland) and MedCalc v. 14.8.1 (MedCalc Software bvba, Ostend, Belgium).

An analysis was performed of respondents’ age structure, education, marital status, nutritional status, visceral obesity, physical activity level, current severity of LUTS, period of current BPH treatment, current pharmacotherapy for BPH, comorbidities, ED, and DSs.

The data collected with regard to DSs and ED were analysed according to age, education level, marital status, alcohol consumption, physical inactivity, comorbidities including obesity, severity of LUTS, treatment with inhibitor 5-α reductase or anticholinergic medications, TURP history, nocturia, ED, or DSs, respectively.

Values of variables were presented as percentages and mean values with SD. Separate groups were compared using the *χ* ^2^ test and *χ* ^2^ test for trend and *t* Student test for independent variables, and post hoc Tukey’s test. The odds ratios for factors influencing ED and DSs were calculated based on the stepwise backward multiple logistic regression analysis. Multicollinearity has been check during the logistic regression procedure based on condition numbers (CN). The rule of thumb with CN greater than 15 has been used to remove correlated factors. A *p* < 0.05 was considered as statistically significant.

## Results

### Characteristics of the surveyed group

The surveyed group was dominated by respondents aged 61–80 years, the medium city dweller, with secondary education, married, and professionally active or pensioner (Table 1).

66.2 % responders declare sedentary life style, 18.8 % frequent alcohol consumption, 29.8 % smoking currently, and 24.5 % had in the past (Table 1).

Obesity according to WHO criteria was diagnosed in 28.9 % responders, and visceral obesity according to IDF criteria was diagnosed in 53.5 % (Table 1). Comorbidities were reported in 83.4 % of the surveyed group. The most common comorbidities were hypertension (53.6 %), coronary artery disease (18.4 %), dyslipidaemia (17.6 %), and type 2 diabetes (16.7 %)—Table 2.

**Table 2:** Severity of lower urinary tract symptoms (LUTS), therapy of benign prostatic hyperplasia (BPH), coexisting diseases, depression, erectile dysfunction, and nocturia in 4,035 patients with BPH

Severity of LUTS before treatment [pts.]	16.8 ± 5.9
Mild [n(%)]	123 (3.0)
Moderate [n(%)]	2,651 (65.7)
Severe [n(%)]	1,261 (21.3)
Severity of LUTS currently [pts.]	10.2 ± 5.7
Mild [n(%)]	1,774 (44.0)
Moderate [n(%)]	1,972 (48.8)
Severe [n(%)]	289 (7.2)
Period of treatment for BPH [n(%)]	
Less than one year	861 (21.4)
1–2 years	1,044 (25.9)
3–5 years.	1,056 (26.2)
More than 5 years.	1,074 (26.5)
Current BPH pharmacotherapy [n(%)]	
Monotherapy	2,052 (50.9)
α_1_-selective adrenergic receptor antagonist (ARA)	1,918 (47.5)
5α reductase inhibitor (I5αR)	134 (3.3)
Polytherapy	1,931 (47.9)
ARA + I5αR	1,623 (40.2)
ARA + I5αR + anticholinergic	308 (7.6)
No pharmacotherapy (patients after TURP)	52 (1.3)
Past TURP [n(%)]	52 (1.3)
Comorbidities	
Coronary artery disease [n(%)]	742 (18.4)
Past myocardial infarction [n(%)]	340 (8.5)
Heart failure [n(%)]	108 (2.7)
Diabetes [n(%)]	669 (16.7)
Past stroke episode [n(%)]	129 (3.2)
Hypertension [n(%)]	2,170 (53.6)
Chronic kidney disease [n(%)]	48 (1.2)
Dyslipidaemia [n(%)]	712 (17.5)
Erectile dysfunction [n(%)]	2,900 (71.9)
Depression symptoms:	904 (22.4)
Mild [n(%)]	840 (20.8)
Moderate/severe [n(%)]	64 (1.6)
Nocturia [n(%)]	2,554 (63.3)

### Medical history of BPH and its treatment

More than half of the patients were treated for BPH longer than 3 years (Table 2). Transurethral resection of the prostate (TURP) was performed in 1.3 % of the surveyed population, and pharmacotherapy was not prescribed to these subjects.

Currently, more than half of patients were on monotherapy, mostly with ARA (47.5 % of surveyed population). Polytherapy was prescribed for 47.9 % of the surveyed population, commonly with ARA and 5αRI (40.2 %). Only 7.6 % of the surveyed population was treated with muscarinic receptor agonists (MRA) as a part of polytherapy (the third drug)—Table 2.

Before starting the therapy for BPH, 65.7 % of surveyed subjects presented moderate and 21.3 % severe LUTS. Current pharmacotherapy was associated with decreased frequency of moderate or severe symptoms by 66 % (Table 2). Nocturia symptoms had been found in 63.3 % surveyed population.

### Erectile dysfunction

ED based on IIEF-5 was diagnosed in 71.9 % of the surveyed population (in all after TURP), and 30.2 % were treated for ED (Table 2).

**Fig. 1:**
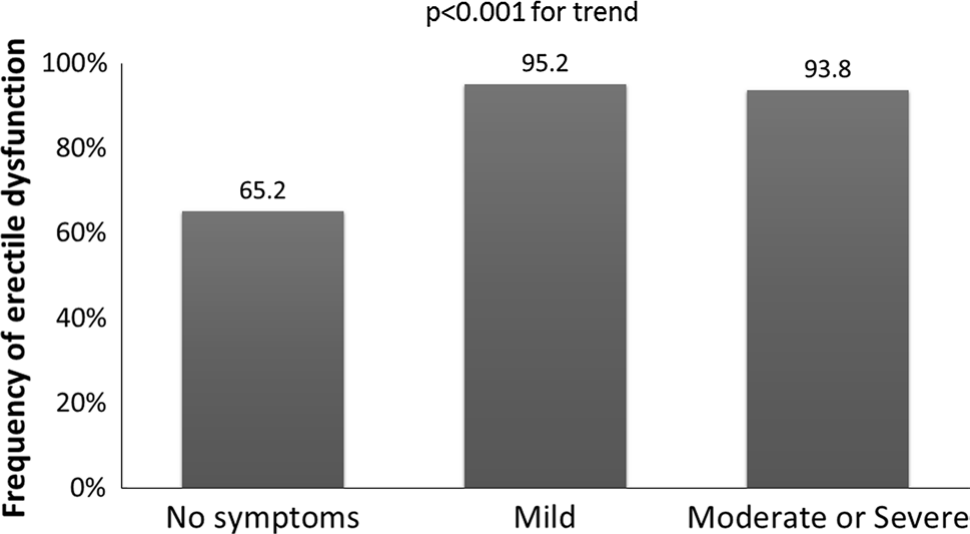
Frequency of erectile dysfunction in relation to the occurrence and severity of depressive symptoms in patients diagnosed with benign prostatic hyperplasia (BPH)

The prevalence of ED in relation to demographic and clinical factors is shown in Table 3. The statistical analysis revealed that ED occurrence was related to increased age, low education level, widowed or single marital status, sedentary life style, abstinence from or infrequent alcohol consumption, comorbidities (such as obesity, visceral obesity, past myocardial infarction or stroke, heart failure, hypertension, diabetes, and chronic kidney disease), length of time of BPH pharmacotherapy, polytherapy for BPH, history of TURP, LUTS severity, nocturia, and occurrence of moderate or severe DSs (Table 3). The association between ED and severity of DSs presents Fig. 1.

**Table 3:** Frequency of erectile dysfunction and depressive symptoms in relation to sociodemographic and clinical factors in 4,035 patients with benign prostatic hyperplasia

	Erectile dysfunction %	Depressive symptoms	
		Mild %	Moderate or severe %
*Age groups*			
≤60 years	56.0	15.8	0
61–80 years	77.2	22.8	2.1
>80 years	100	19.2	3.8
Education			
Primary	85.4	52.0	7.2
Vocational	70.1	20.1	2.0
Secondary	72.3	18.5	0.7
Higher	68.2	12.9	0
*Marital status*			
Married	67.3	15.2	0.7
Widowed/single	89.3	36.6	4.1
*Nutritional status*			
Normal weight	63.8	16.2	1.4
Overweight	68.3	16.9	0.9
Obese	83.1	30.9	3.1
*Visceral obesity*			
Yes	79.1	23.7	1.7
No	63.7	17.7	1.5
*Physical activity*			
<30 min a day	76.2	24.9	1.9
30–60 min a day	65.2	12.5	1.2
>60 min a day	58.7	14.1	0
*Cigarette smoking*			
Ever smokers	72.3	21.5	1.5
Non-smokers	71.4	20.2	1.6
*Alcohol consumption*			
Frequent	59.7	23.2	4.2
Seldom/abstinent	74.7	20.3	1.0
*Current severity of LUTS*			
Mild	48.5	7.7	0
Moderate	89.2	26.0	1.6
Severe	97.2	66.4	11.1
*Period of BPH treatment*			
<1 year	29.4	6.0	0
1–2 years	68.1	14.6	0.8
3–5 years	88.6	26.5	2.3
>5 years	93.2	33.1	3.0
*BPH treatment*			
Monotherapy	55.8	10.3	1.2
Polytherapy	88.5	31.7	2.0
α-1 adrenergic receptor antagonist (ARA)	54.4	10.0	0.8
5-α reductase inhibitor (I5αR)	75.8	14.9	6.0
ARA + I5αR	89.4	30.8	1.0
ARA + I5αR + muscarinic receptor agonist	89.1	33.8	6.5
No pharmacotherapy (past TURP)	100	44.1	7.7
*Comorbidities*			
Coronary artery disease			
Yes	72.4	31.8	3.2
No	71.8	18.3	1.2
*Past myocardial infarction*			
Yes	85.9	43.5	5.9
No	70.6	18.7	1.2
*Heart failure*			
Yes	96.3	59.3	14.8
No	71.2	19.8	1.2
*Diabetes*			
Yes	79.6	35.9	4.2
No	70.3	17.8	1.1
*Past stroke episode*			
Yes	96.9	31.0	6.2
No	71.1	20.5	1.4
*Hypertension*			
Yes	74.7	19.7	1.1
No	68.6	22.1	2.1
*Chronic kidney disease*			
Yes	100	66.7	25.0
No	71.5	20.3	1.3
*Depression symptoms*			
Moderate or severe	97.1	–	–
Mild	89.8	–	–
No symptoms	62.7	–	–
*Erectile dysfunction*			
Yes	–	27.5	2.1
No	–	7.6	0.4
*Nocturia*			
Yes	79.3	27.4	2.2
No	59.2	9.4	0.5

A backward model of multivariate logistic regression analysis demonstrated that the most important factors associated with the occurrence of ED were as follows: past cardiovascular episodes, heart failure, and depression. Therapy with inhibitors of 5-alpha reductase had negative effects, while the use of anticholinergic drugs was related to less frequent ED. Among factors that reduce the prevalence of ED were as follows: being married, higher education level, and frequent alcohol consumption (Fig. 5).

### Depressive symptoms

DSs were found in 22.4 % of the surveyed population, including mild symptoms in 20.8 % and moderate/severe symptoms in 1.6 %. The prevalence of DSs in relation to demographic and clinical factors is shown in Table 3.

**Fig. 2:**
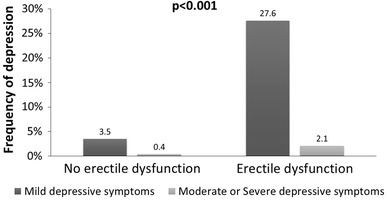
Frequency of depressive symptoms in relation to prevalence of erectile dysfunction

**Fig. 3:**
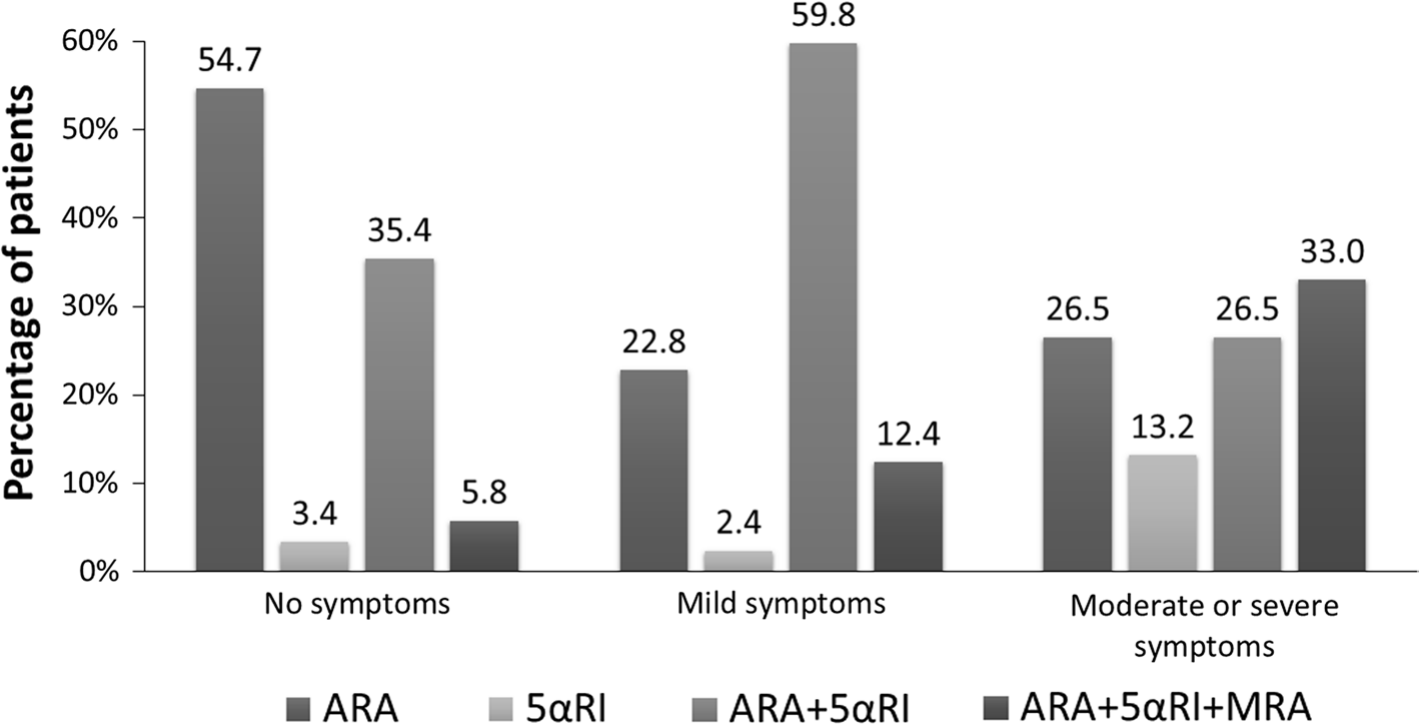
Structure of pharmacotherapy for BPH in relation to the occurrence of depressive symptoms in 4,035 patients diagnosed with BPH

**Fig. 4:**
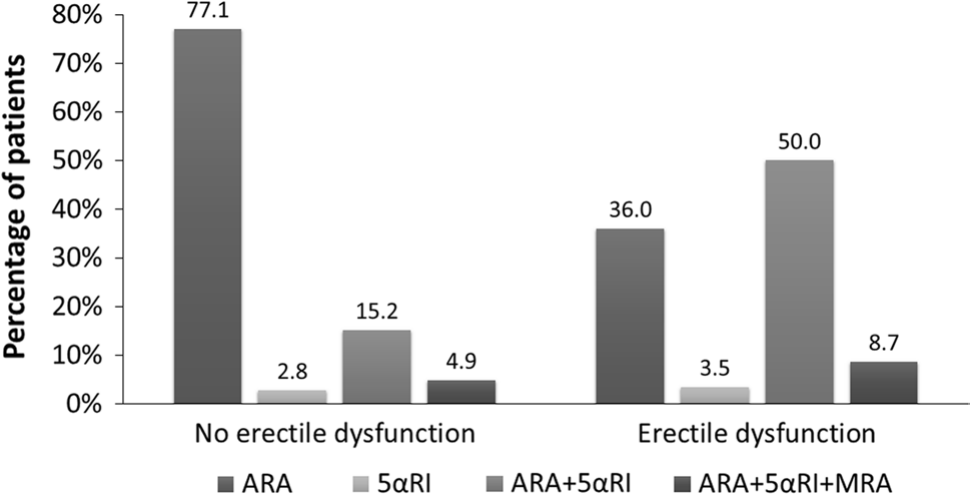
Structure of pharmacotherapy for BPH in relation to the occurrence of erectile dysfunction in 4,035 patients diagnosed with BPH

Statistical analysis revealed that DSs occurrence and severity were related to older age, lower education level, widowed or single marital status, sedentary life style, comorbidities (such as obesity, visceral obesity, coronary artery disease, past myocardial infarction or stroke, heart failure, diabetes and chronic kidney disease), longer time of BPH pharmacotherapy, polytherapy, history of TURP, LUTS severity, nocturia occurrence, and ED (Table 3). The association between DSs and ED presents Figs. 2, 3 and 4.

**Fig. 5:**
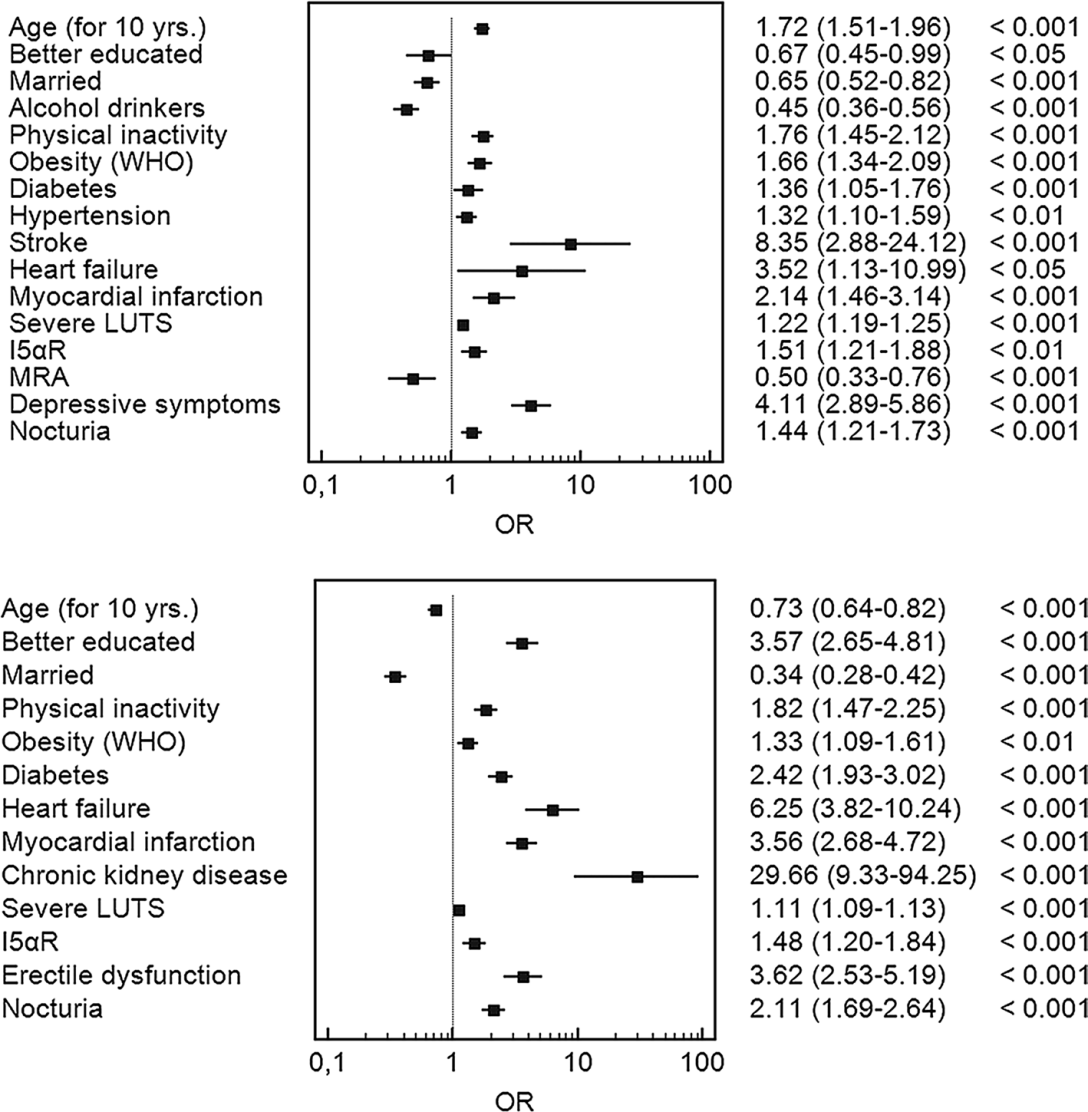
Factors influencing the incidence of erectile dysfunction (*upper*) and depressive symptoms (*lower*) in the group 4,035 patients diagnosed with benign prostatic hyperplasia. Results of stepwise multivariate backward logistic regression

In the model of backward multivariate logistic regression analysis, it was found that the most important factors associated with the occurrence of DSs are as follows: chronic kidney disease, heart failure, cardiovascular episodes, diabetes, ED, the use of 5αRI inhibitors, and the severity of LUTS. Secondary or higher or educational level and being married were the only factors reducing the prevalence of DSs (Fig. 5).

## Discussion

This study showed that 22.4 % of patients treated for BPH had DSs, and 71.9 % ED. The occurrence of both DSs and ED was associated with comorbidities (heart failure, coronary artery disease, past myocardial infarction, diabetes, and obesity), as well as with the severity of LUTS, nocturia occurrence, current BPH pharmacotherapy (5αRI), solitary dwelling, and sedentary life style. Some comorbidities were more strongly affecting ED (hypertension, past stroke), although in others, it was the prevalence of DSs (chronic kidney disease). Furthermore, there was a bidirectional association between DSs and ED; however, the effect of the occurrence of DSs on ED [OR 4.14 (95 % CI: 2.91–5.90)] was more manifested than vice versa [3.06 (95 % CI: 2.15–4.36)].

The incidence of DSs in patients with BPH was analysed in few studies. The prevalence of DSs in the American population with LUTS, assessed on the basis of Geriatric Depression Scale (GDS), was similar to that in our study (17.2–22 % vs. 22.4 %) [23, 24]. In accordance with our results, Johnson et al. observed an association between DSs and LUTS severity. However, they suggested that depressed patients report elevated symptoms [23]. The association between DSs and BPH was also confirmed by results obtained in the Taiwanese population, showing development of depression in 2 % of patients diagnosed with BPH during a year, 1.87 times more frequently than in a comparable cohort of men without BPH [24]. Also, little is known about factors that predispose to development of depression in patients with BPH. In Hong Kong and Chinese populations, the factors influencing the prevalence of DSs in patients with BPH were loneliness (widowed, divorced, single status), smoking, the burden of coronary heart disease history, the use of corticosteroids, and moderate or severe LUTS [25, 26]. Loneliness, CVD, and LUTS severity were also factors influencing DSs occurrence in our study. Additionally, Rom et al. [27] showed a relationship between LUTS and DSs in men, using methodology similar to ours (IPSS and BDI). The association between LUTS and DSs is also indirectly confirmed by the studies showing that urinary urgency, frequency, and nocturia have a significant impact on men’s lives, degree of worry, interference with psychological well being [28, 29].

Our most relevant finding is the association between BPH pharmacotherapy and DSs occurrence. We demonstrated that the use of a 5-αRI is associated with a 1.52-fold higher prevalence of DSs. It is in line with a study showing that the use of finasteride, a 5-αRI might induce development of depression [30]. Furthermore, we found that DSs occur very commonly (51.8 %) in patients who underwent TURP, more often than in those receiving combine therapy (31.7 %) even on three drug therapy (with MRA). Contrary, prospective studies show that the level of depression and anxiety decreases after TURP, along with the reduction in LUTS severity [31, 32]. It may be related to increased prevalence of ejaculation disorders after TURP, much higher than in patients treated with 5-αRI [33].

Among other factors explaining the prevalence of DSs in our study population were frequent alcohol consumption (more than three times per week) and nutritional status—mostly obesity. Similar results were obtained previously by other researchers. It was shown that alcohol consumption increases the risk of developing depression [34, 35]. Also, sedentary lifestyle (physical inactivity) and obesity are associated with the prevalence of DSs [36, 37].

Our study demonstrates that at least 90 % of subjects with DSs have ED, the most common sexual dysfunction among elderly men. The impact of this category of sexual dysfunction on sexual activity is significant, and it is obvious that these men seek treatment [38]. In the current study, 30.2 % of patients with ED were treated for this reason.

In line with previously published data, we demonstrated increased prevalence, with advancing age, of ED. Data from the Massachusetts Male Ageing Study showed that the annual incidence of ED was increasing from 12 per 1,000 men aged 40–49 years to 46 per 1,000 men in 60–69 years of age [31]. In the cologne male survey, the prevalence of ED was 19.2 %, with a steep age-related increase (2.3–53.4 %) and strong association with comorbidities, e.g. hypertension, diabetes, pelvic surgery, and LUTS [40].

It should be stressed that our study demonstrates that the therapy with 5-αRI has a deteriorative effect on ED, while the use of MRA is beneficial in multiple regression analysis. The beneficial effect of MRA did not appear in simple regression, as this group of drugs is prescribed in patients with severe LUTS. Such aspect has not been studied yet. Hypothetically muscarinic receptor stimulation may facilitate erection and ejaculation demonstrated in rats with a transected spinal cord [41]. This observation requires further studies.

ED shows a strong correlation with quality of life. The prevalence of ED increases with the occurrence of concomitant conditions such as heart disease, hypertension, depression, negative mood, problems with relationships, or just inadequate sexual experience [39, 42]. Greenstein et al. [43] found a correlation between the severity of coronary artery disease and ED. In the Massachusetts Male Ageing Study, after adjustment for age, a higher probability of impotence was directly correlated with heart disease, hypertension, diabetes, depression, and their associated medications [44]. The relationship between ED and comorbidities was similar.

Unhealthy lifestyle, including physical inactivity, smoking, and obesity, are among well-known factors affecting the occurrence of ED [45, 46]. However, not all behaviours have deleterious effects. Multivariate logistic regression analysis of our data revealed that physical inactivity and obesity, but not frequent alcohol consumption (more than three times a week) increased the risk of ED. It was already demonstrated that consumption of eight or more drinks/week significantly reduced the risk of ED [47].

Our study has limitations resulting from methodology—cross-sectional design. It should be noted, that the diagnosis of BPH was based on clinical examination (LUTS, DRE, and sonography). Therefore, we cannot exclude that some patients were suffering from undiagnosed prostate cancer. However, due to lack of diagnosis of the cancer, it could not be a factor affecting the level of depression. It should be stressed, that the survey did not include men with diagnosed mental disorders, including depression.

Therefore, the prevalence of depressive syndrome is Polish BPH patients is underestimated. The recently performed PolSenior study revealed that only 13.6 % of elderly with DSs were diagnosed with depression [48]. Taking into account this data, we may estimate the prevalence of DSs among patients with BPH is approximately 26 %.

## Conclusion

Prevalence of depressive symptoms in patients diagnosed with BPH is associated with severity of LUTS, erectile dysfunction, nocturia, BPH pharmacotherapy (5αRIs), sedentary life style, and comorbidities including obesity.
